# Case Report: Coronary artery stent infection with mycotic aneurysm secondary to tricuspid valve infective endocarditis

**DOI:** 10.12688/f1000research.19067.1

**Published:** 2019-06-12

**Authors:** Mejdi Ben Messaoud, Nidhal Bouchahda, Marouane Mahjoub, Badii Hmida, Zohra Dridi, Habib Gamra

**Affiliations:** 1 Cardiology A Department, Fattouma Bourguiba University Hospital, Monastir, Monastir, Monastir, 5000, Tunisia; 2 Radiology Department, Fattouma Bourguiba University Hospital, Monastir, Monastir, Monastir, 5000, Tunisia

**Keywords:** endocarditis, stents, infection, coronary aneurysm, acute coronary syndrome

## Abstract

Coronary artery stent infection with mycotic aneurysm is a rare life-threatening complication following coronary angioplasty, usually requiring surgical intervention. Reaching and confirming the diagnosis remains the most challenging aspect of this complication. We describe an unusual case of bare metal stent infection and coronary artery aneurysm in the setting of tricuspid valve infective endocarditis, resulting in ST elevation myocardial infarction, with a favorable outcome after primary angioplasty and antibiotic therapy. In the current era of growth of coronary stent implantation, it’s important for clinicians to consider and to prevent such potentially fatal events. The diagnosis process remains difficult and requires the association of multiple clinical, biological and imaging parameters. Although treatment modalities tend to favor surgery, we showed that coronary angioplasty could be a successful alternative solution.

## Introduction

Infectious complications following percutaneous cardiac interventions are known but not very common. Coronary stent implantation is associated with complications, though rare, such as stent infection or coronary aneurysms resulting from several mechanisms, and needing special investigations for their diagnosis and management ^1, 2^. Simultaneous occurrence of coronary stent infection and mycotic aneurysm has been described in a few cases as an unusual life-threatening complication following coronary angioplasty ^3^. The majority of the few reported cases had an unfavorable outcome ^4^, with the majority treated surgically. 

We report a rare case of ST elevation myocardial infarction due to bare metal stent infection and coronary artery aneurysm in the setting of tricuspid valve infective endocarditis. The management and outcome of our patient was quite different from what was reported in the literature.

It is important for the interventional cardiologist, to remember that such complication might occur and should be prevented. If it does occur however, its treatment and outcome represent a challenge for clinicians.

## Case

**Figure 1.**
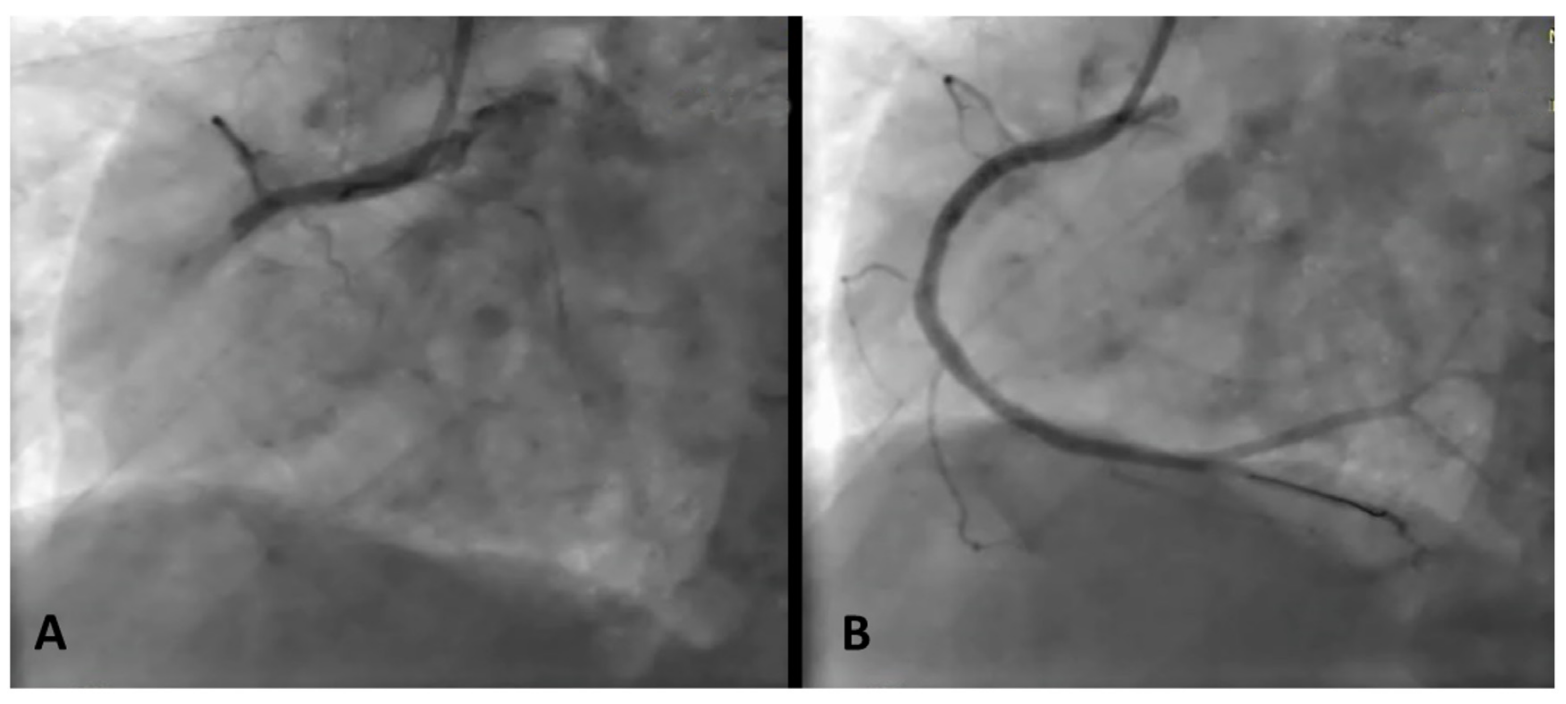
Coronary angiogram images before ( **A**) and after ( **B**) the first primary angioplasty of the right coronary artery.

**Figure 2.**
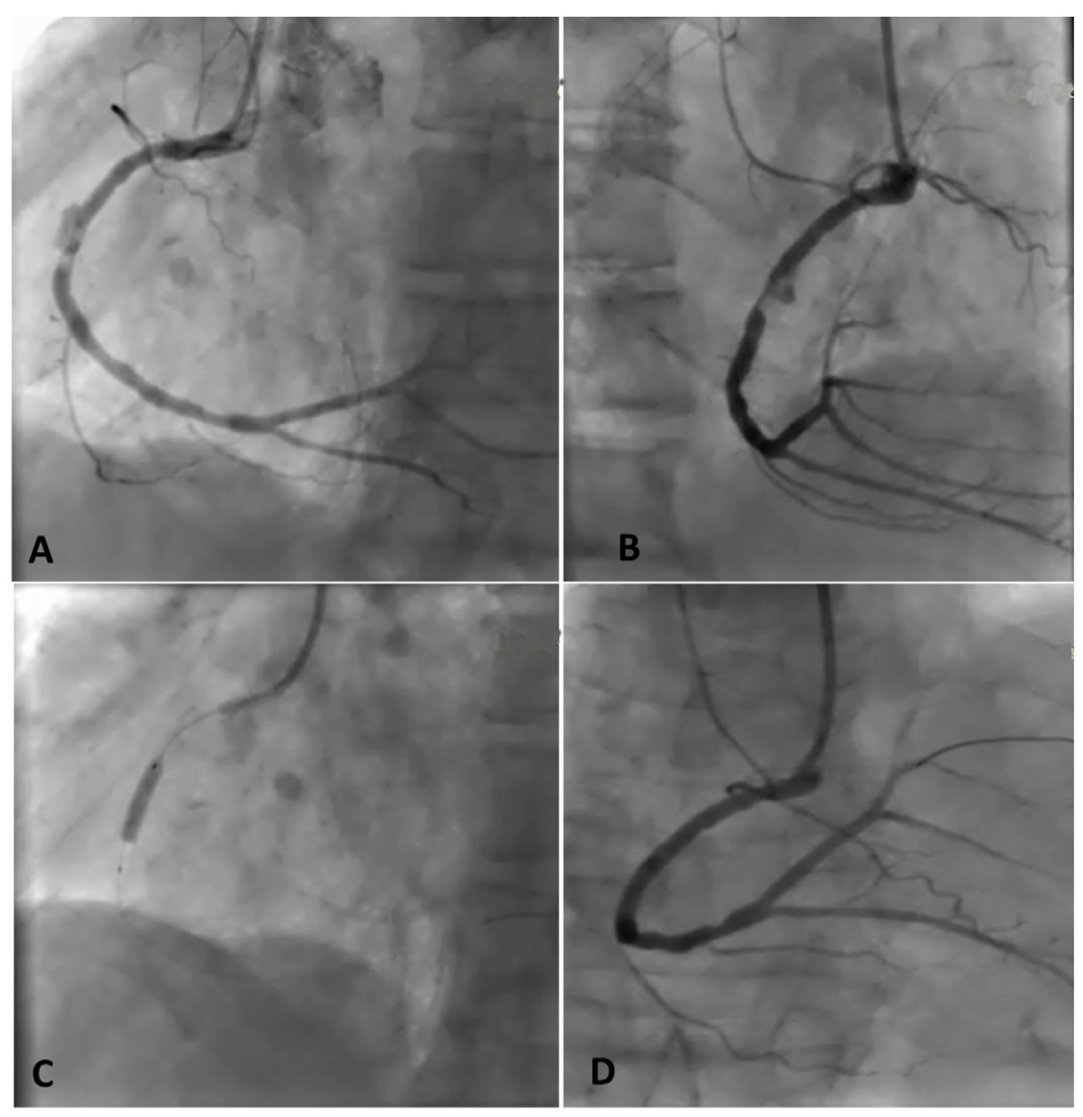
Coronary angiogram images of the second primary angioplasty: **A** and **B** show the intrastent thrombus with mycotic aneurysm of the right coronary artery. **C** and **D** show the right coronary artery angioplasty with an overlapping bare metal stent.

**Figure 3.**
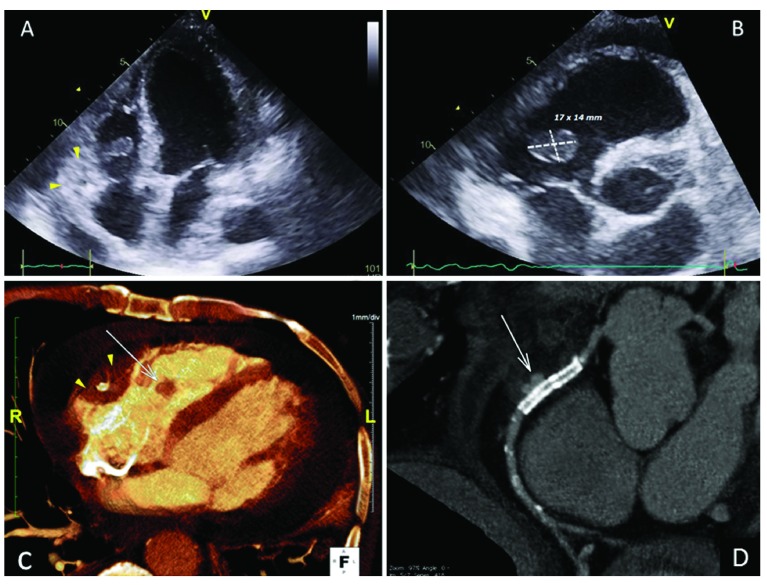
Imaging showing tricuspid valve vegetation (arrow) with hematoma around the right coronary artery (arrowhead) and pericardial effusion in transthoracic echocardiography ( **A** and **B**) and cardiac computed tomography scan ( **C**). **D**: computed tomography coronary angiography showing the right coronary artery aneurysm excluded by the overlapping stent.

A 71-year-old North African retired male patient with a history of smoking (40 pack-years) and no known medical or surgical history, was referred to our center in October 2017, from the emergency department, for inferior ST elevation myocardial infarction with complete atrioventricular block. He underwent a successful primary angioplasty of the right coronary artery (RCA) with a bare metal stent ( Figure 1). A temporary cardiac pacing via the right femoral vein was performed for 24 hours and the patient was discharged at day 5. Initial and controlled laboratory tests during hospitalization were normal except elevated troponin at admission. One week later, the patient was referred again to our center from the emergency department for persistent chest pain with a new right bundle branch block. An urgent coronary angiogram showed a thrombus with an aneurysm on the distal part of the RCA stent and a Thrombolysis In Myocardial Infarction (TIMI) 3 flow (normal flow) ( Figure 2A and 2B). An additional angioplasty using a second bare metal stent overlapping with the previous one was successfully performed with exclusion of the aneurysm and disappearance of the thrombus. ( Figure 2B and 2C). The initial physical exam in the coronary care unit immediately after angioplasty found a mild fever at 38°C with a systolic tricuspid regurgitation murmur. Echocardiography showed vegetation (17 × 14 mm) at the level of the tricuspid valve with a moderate tricuspid regurgitation and a moderate pericardial effusion ( Figure 3A and 3B). Transesophageal echocardiography confirmed the same findings and ruled out a patent foramen ovale or any involvement of the other valves. Laboratory tests (which were normal at the previous admission) showed a marked elevation of white blood cells count (24,000 E/mm3, normal value < 11,800 E/mm3), C reactive protein (186 mg/L, normal value < 6 mg/L), and liver enzymes (Alanine transaminase (ALT) at 215 IU/L and Aspartate aminotransferase (AST) at 165 IU/L, normal value respectively < 35 IU/L and < 40 IU/L) associated with acute renal failure (serum creatinine at 148 mmol/L, normal value < 105 mmol/L) and hemolytic anemia (Hemoglobin 8 g/DL, normal value > 13 g/DL). Blood cultures were positive to negative coagulase *Staphylococcus*. The diagnosis of infective endocarditis of the tricuspid valve with RCA mycoticaneurysm at the site of stent implantation was strongly suspected. Cardiac CT scan confirmed the vegetation in the right ventricle with hematoma around the RCA and pericardial effusion ( Figure 3C). It also showed a mycotic aneurysm of the RCA which was excluded by the overlapping stent ( Figure 3D). Following a thorough search for the underlying cause, we concluded that the temporary cardiac pacing was the most likely origin of the tricuspid valve infective endocarditis. Antibiotic therapy (vancomycin 30 mg/Kg/day i.v. in 2 doses, rifampicin 900 mg orally in 3 divided doses and gentamycin 3 mg/Kg i.v once daily) was provided resulting in total regression of the infection symptoms, the tricuspid vegetation and the pericardial effusion. The patient was discharged from the hospital after six weeks of antibiotic therapy. The patient has completed one-year of follow-up with repeated echocardiography and blood tests, all of which were ordinary.

## Discussion

Stent infection can occur early (less than 10 days) or late (10 days or longer) following coronary angioplasty ^4^ and is frequently diagnosed in the context of an acute coronary syndrome due to stent thrombosis or coronary artery aneurysm. The diagnosis of coronary stent infection is confirmed based on the criteria proposed by Dieter ^1^. The diagnosis is definitive when confirmed by autopsy or surgical material examination if at least three of the following criteria are present: coronary stent implantation during the last 4 weeks; repeated interventions through the same arterial sheath; fever > 38°C, documented bacteremia, leukocytosis without evident etiology; acute coronary syndrome; or positive cardiac imaging. Mycotic coronary aneurysms may result from different mechanisms including direct bacterial invasion in the setting of infective endocarditis, injury caused by immune complexes, arterial trauma and vasa vasorum embolic occlusion. Other mechanisms such as immuno-compromised status and congenital cardiovascular defects might be involved ^2^. Following testing for all these mechanisms in our patient we strongly suspected the infective endocarditis as the main etiology of the right coronary stent mycotic aneurysm. The majority of the similar reported cases were associated with a fatal outcome ^4^. In few cases, like in ours, peri-coronary hematoma with pericardial effusion can be present and it may be due to the coronary aneurysm leak or rupture ^5^. Irrespective of time of presentation, the majority of stent infection with coronary aneurysm must be treated by surgical extraction of the stent with aneurysm repair when indicated and with simultaneous intravenous antibiotic therapy for at least 4 weeks ^6^. In our case, the follow up at 1 year didn’t show any signs of relapse. Consequently, the patient was not referred to surgery.

One limitation of this report relates to the mycotic nature of the stent aneurysm which can only be confirmed by tissue analysis. This was not possible since the patient did not have surgery nor a postmortem autopsy. However, the diagnosis was highly probable based on the clinical, biological and imaging parameters.

In conclusion, coronary stent infection in the setting of infective endocarditis is rare but must be considered whenever a patient develops fever and chest pain after stent implantation. Treatment modalities tend to favor surgery, but in our case, we showed that coronary angioplasty and prolonged antibiotic therapy may be sufficient. To prevent such complications, adherence to aseptic precautions and treatment of pre-existing infections are of paramount importance.

## Consent

Written informed consent for publication of their clinical details and/or clinical images was obtained from the patient/parent/guardian/relative of the patient.

## Data availability

### Underlying data

All data underlying the results are available as part of the article and no additional source data are required.
