# Supplementary material for: Case Report: Coronary artery stent infection with mycotic aneurysm secondary to tricuspid valve infective endocarditis
Source: F1000Res. 2019 Jun 12;8:853. [Version 1] doi: 10.12688/f1000research.19067.1 (PMC6795233; doi:10.12688/f1000research.19067.1)
Supplement: Peer review history

# Peer review: Reviewer response for version 1

**DOI:** 10.5256/f1000research.20896.r49820

**Published:** 2019-10-15

**Authors:** Marouane Boukhris

**Affiliations:** 1 Cardiology Department, Centre Hospitalier de l'Université de Montréal, Montréal, Canada

In the current manuscript, the authors reported a rare infective complication following percutaneous coronary intervention. They underlined the difficulty of the diagnosis, particularly when neither surgery nor autopsy is performed. 

 Generally, the manuscript is well written and the topic is quite interesting for the reader. The paper seems suitable for indexing in its actual version.

I confirm that I have read this submission and believe that I have an appropriate level of expertise to confirm that it is of an acceptable scientific standard.

# Peer review: Reviewer response for version 1

**DOI:** 10.5256/f1000research.20896.r49816

**Published:** 2019-07-10

**Authors:** Nicolas Amabile

**Affiliations:** 1 Institut Mutualiste Montsouris, Paris, France

In this paper, Ben Messaoud and colleagues reported a very unusual case of a patient who developed a subacute stent thrombosis in the setting of a tricuspid acute endocarditis. The ST was associated with an image of aneurysm on the distal edge of the stent. Although there are other causes of coronary artery aneurysm (including iatrogenic media disruption, stent fracture or severe stent malapposition) that could appear after initial thrombus dissolution, the authors concluded to a mycotic aneurysm. This conclusion makes sense regarding the clinical context, CT scan data and subsequent evolution following antibiotics therapy. The main limitation of the report is the lack of pathology analyses and was acknowledged by the authors. 

 The manuscript in this current version raises the following issues:

1. What was the antiplatelet drug regimen of the patient after the first STEMI ? What about the patient compliance?
2. The CT scan initial data could be better explained and a cross sectional image of the RCA showing the hematoma might be a nice addition.
3. Do the authors have a 1 year CT scan or angio to illustrate the aneurysm disappearance (or no)?
4. What is the authors opinion about implanting a new device in an actively infected stent? 

I confirm that I have read this submission and believe that I have an appropriate level of expertise to confirm that it is of an acceptable scientific standard.

# Response

**Published:** 2019-07-10

**Authors:** Mejdi Ben Messaoud

**Affiliations:** Fattouma Bourguiba University Hospital, Monastir, Tunisia

I would like to thank Dr Amabile for his precious comments. 

The answers for his questions are as follows:

1. The antiplatelet therapy included aspirin 100 mg once a day and Clopidogrel 75 mg once a day. There was no history of DAPT discontinuation.
2. I agree that a cross sectional image of the RCA showing the hematoma might be a nice addition.
3. No CT scan was performed after 1-year follow up. The follow up was based on clinical exam, biological tests and echocardiography.
4. We think that implanting a new device in an actively infected stent is not recommended, but in our case, we have performed an ad-hoc angioplasty because the diagnosis of stent infection was not confirmed.
